# The Effect Dry Cupping Therapy at Acupoint BL23 on the Intensity of Postpartum Low Back Pain in Primiparous Women Based on Two Types of Questionnaires, 2012; A Randomized Clinical Trial

**Published:** 2014-04

**Authors:** Marzieh Akbarzadeh, Mehrnoush Ghaemmaghami, Zahra Yazdanpanahi, Najaf Zare, Amir Azizi, Abdolali Mohagheghzadeh

**Affiliations:** 1 Community Based Psychiatric Care Research Center, Department of Midwifery, School of Nursing and Midwifery, Shiraz University of Medical Sciences, Shiraz, Iran;; 2 Department of Midwifery, School of Nursing and Midwifery, Shiraz University of Medical Sciences, Shiraz, Iran;; 3 Department of Bio-Statistics, Infertility Research Center Shiraz University of Medical Sciences, Shiraz, Iran;; 4 Department of Pharmacognosy, Shiraz University of Medical Sciences, Shiraz, Iran

**Keywords:** Dry Cupping, Acupuncture Point BL23, Low Back Pain, Postpartum

## Abstract

**Background:** Continuous low back pain is associated with the symptoms of the pregnancy period. In spite of the improvement of low back pain within 6 months after the delivery, some women may develop chronic problems. This study aimed to investigate the effect of dry cupping therapy at BL23 point on the intensity of low back pain in primiparous women.

**Methods:** In the present randomized clinical trial, 100 samples were randomly allocated to either the cupping therapy or the control group (each containing 50 subjects). Cupping therapy was performed for 15-20 minutes every day up to 4 consecutive times. Visual Analogue Scale (VAS) and short-form McGill pain questionnaire were completed by the two groups before the intervention and immediately, 24 hours, and 2 weeks after that. Then, the data were entered into the SPSS statistical software (v. 16) and analyzed using chi-square test and repeated measures ANOVA.

**Results:** According to VAS, the mean intensity of low back pain in the cupping therapy group decreased from 7.8±2.7 before the intervention to 3.7±1.8, 2.5±1.7, and 1.4±1.4 immediately, 24 hours, and 2 weeks after the intervention, respectively. Besides, these measures were respectively obtained as 31.8±10.8, 9.0±6.7, 7.5±6.6, and 3.6±4.1 in the short-form McGill pain questionnaire. According to repeated measures ANOVA, a significant difference was observed among the various stages of follow-up (P=0.01).

**Conclusion:** The study results showed cupping therapy to be effective in sedation of pain. Thus, it can be used as an effective treatment for reducing the low back pain.

**Trial Registration Number:** 2013072611944N3

## Introduction

Back pain has been reported in almost 70% of the women in the reproductive age.^1^ Moreover, this pain is detected in 50-80% of the women during pregnancy.^2^^,^^3^ and can clinically lead to long-term pain and disability after the delivery.^4^ In the first month after the delivery, the prevalence of this pain has been reported to be 35%, but it improves afterwards.^5^^,^^6^ In fact, low back pain is the most prevalent pain during pregnancy which is considered as a serious problem by one third of the pregnant women.^7^ In one study, 61.8% of the women reported relatively severe pain during pregnancy. In addition, 9% of the women were completely disabled by the pain.^8^^,^^9^ In some women, this pain spontaneously disappears after the sixth month of the puerperium period, while it chronically continues in other ones. One study found that the prevalence of back pain would decrease to the pre-pregnancy level up to 2 years after the delivery (18%).^9^

Overall, most of the information about pelvic girdle pain has been gathered from the western countries. In fact, a limited number of studies have been conducted on the pregnancy-related pelvic girdle pain and low back pain in eastern and Middle Eastern countries. The results of an Iranian study evaluating the difference between pelvic girdle and low back pain during pregnancy showed that 91 (28%), 43 (13.2%), and 27 (8.3%) women suffered from pelvic girdle pain, low back pain, and both pains, respectively. In addition, Visual Analogue Scale (VAS) revealed the intensity of pain to be 5.6 among the women with pelvic girdle pain. In that study, one out of every two women suffered from pelvic girdle pain and the prevalence of this pain was two folds higher than that of low back pain. This implies that pelvic girdle pain is a major health problem among the Iranian pregnant women and requires more attention on the part of the authorities of the health and treatment system.^10^

Back pain is generally related to the changes in body weight and mobility resulting from carrying the fetus during the pregnancy period. Increase in weight during pregnancy directs the center of the gravity of the body forward and, consequently, increases the pressure imposed on the lumbar spine. This eventually leads to symphysis pubis dysfunction.^11^ Yet, treatment based on continuous exercises is important for management of postpartum pain and is accompanied by improvement of function, health related quality of life, and physiological status.^12^ In general, various methods, including resting, topical hot or cold packs, physiotherapy, nerve stimulation through skin, massage, acupuncture, aromatherapy, relaxation, herbal plants, and support belts for postural stability, have been recommended for management of pain during pregnancy.^13^ In addition to the traditional medicine, acupuncture as an alternative medicine has been proved to be quite effective in management of low back pain.^14^ In a study, 61% of health care providers used the Complementary and Alternative Medicine (CAM), such as massage therapy (61.4%), acupuncture (44.6%), relaxation (42.6%), yoga (40.6%), and chiropractic (36.6%) to treat low back pain in pregnancy. Cupping therapy is one of the CAM branches applying to most pain conditions as a traditional medical technique of European, Asian, and Middle Eastern cultures. It is in fact a type of physical therapy which is applied by the specialists of acupuncture or other individuals.^15^ This method improves the subcutaneous blood flow and, as a result, stimulates the autonomous nervous system and reduces the pain.^16^ Moreover, dry cupping therapy involves stimulation of the skin by suction. In this method, a partial vacuum is produced by heat production within the cupping glass after it is applied to the skin. With dry or fire cupping, the cups are applied to the intact skin. In fact, cupping is applied to increase the local blood and lymphatic circulation and to relieve painful muscle tension.^17^^,^^18^ In this study, BL23 point or Shenshu was selected for cupping therapy. This point is located 1.5 cun lateral to the posterior midline, on the level of the lower border of the spinous process of the second lumbar vertebra, thereby providing the opportunity for appropriately placing the cups on a flat space. This point has been utilized in treatment of pain syndromes, such as swelling of low back and knees, genital pain, and gynecological disorders including infertility.^19^

Considering the high prevalence of this pain among the Iranian women and the gap in the available clinical trials on cupping therapy in Iran and the world, the present study attempts to evaluate the effect of dry cupping therapy at the BL23 point on the intensity of low back pain in primiparous women according to VAS and short-form McGill pain questionnaire. It also aims to link the obstetrics and gynecology sciences to the traditional medicine using reliable articles. 

## Materials and Methods

**Figure 1:**
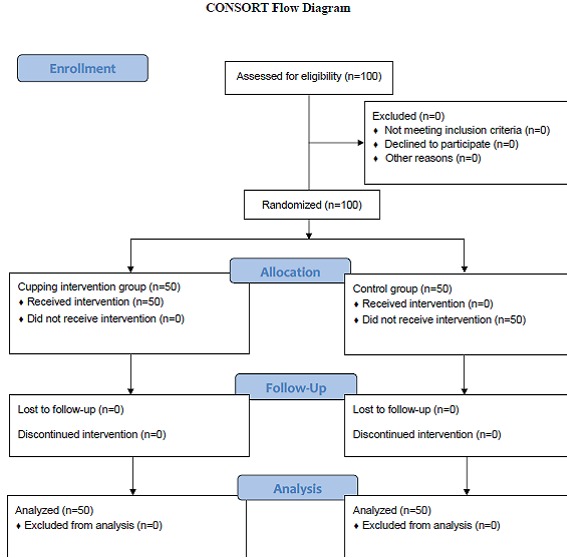
Consort flow diagram of the study participants

The present randomized clinical trial was approved by the Ethics Committee of Shiraz University of Medical Sciences (No. 2013-144). The study was conducted in the postpartum ward of the selected educational-treatment center of Shiraz University of Medical Sciences (Hafez Hospital) in 2012. According to the statistical consultation and using the sample size formula n= [(σ12+σ22)(zα/2+zβ)2]/D2 a 100-subject sample size (50 subjects in each group) was selected for the study. The subjects were selected through purposive sampling. In case an individual did not have the inclusion criteria, she was removed and replaced by the next one. The flow diagram of the participants is shown in figure 1. Then, randomization was performed using the table of random numbers. The inclusion criteria of the study were being between 18 and 40 years old, having at least middle school degree, not suffering from any physical or mental disorders, such as vertebral fracture, disc herniation, acute inflammation, and deep vein thrombosis, during the study, living in Shiraz, being willing to take part in the study, and signing written informed consents. Pain diagnosis was based on the patients’ medical history.

In this study, both interventions were carried out after 8 hours of labor. Cupping therapy was performed by the researcher for 15-20 minutes every day up to 4 consecutive times in the hospital. However, the control group received routine care and was referred to a specialist in case of severe pain. The protocol for performing cupping therapy was as follows: the skin overlying the low back muscle (acupoint: BL23) was disinfected, the vacuum cups (size 75 and 100 cm) were applied, and the air within the cups was rarefied by alcohol and small cotton balls. The intervention was done in retaining model and the cupping glasses were removed after 5 to 15 minutes by pressing one side of the skin with finger to release the vacuum slowly. Then, the participants of both groups completed the VAS and short-form McGill pain questionnaire before and immediately, 24 hours, and 2 weeks after the intervention. VAS, a scale with 10 numbers, is interpreted as follows: No pain (0), mild (1 to 3), moderate pain (4 to 6), severe pain (7 to 9), and the worst pain possible (10).^20^ The validity and reliability of this scale have been confirmed in the study by Molazem et al. Also, studies have reported the Cronbach’s alpha formula coefficient of the scale to be 0.80.^21^

McGill pain questionnaire has been introduced as a reliable instrument for assessment of pain.^22^ However, because it takes a long time for the clients to complete the questionnaire, its short form was designed.^23^ The short form of this questionnaire consists of 15 sensory (11 questions) and emotional (4 questions) items and the patients determine their quality of pain by selecting one of the options of none, mild, average, and severe.^24^ The validity and reliability of the scale in the current research is based on the study by Adelmanesh et al. reporting Cronbach’s alphas of 0.951, 0.832, and 0.840 for sensory, emotional, and total scores, respectively.^25^

After all, the data were entered into the SPSS statistical software (v. 16). After elimination of time and group effects, the mean scores of pain were compared before and after the intervention using repeated measures ANOVA. Besides, chi-square test was employed in order to investigate the demographic characteristics of the two groups. All the statistical tests were performed considering CI=95% and α=0.05. 

## Results

The study results revealed no significant difference between the two groups regarding the demographic variables, such as mother’s age (P=0.064). Moreover, the mean intensity of pain in VAS was 7.8±2.7 and 7.6±2.7 in the cupping therapy and the control group, respectively, but according to the results of repeated measures ANOVA, the difference was not statistically significant (P=0.1). This was also the case regarding the short-form McGill pain questionnaire (31.8±10.8 vs. 31.8±9.8; P=0.1). Immediately after the intervention, however, the mean intensity of low back pain was 3.7±1.8 in the cupping therapy group and 6.4±2.3 in the control group and the results of repeated measures ANOVA revealed a significant difference between the two groups in this regard (P=0.001). The corresponding measures were respectively obtained as 6.7±9.0 and 29.2±8.0 in the short-form McGill pain questionnaire and the reduction was statistically significant. Also, a significant difference was observed between the two groups regarding the mean intensity of low back pain 24 hours and 2 weeks after the intervention (P<0.01). Using VAS 24 hours after the intervention, the mean intensity of pain was 2.5±1.7 and 5.0±2.0 in the cupping therapy and the control group, respectively. Also, it was measured as 1.4±1.4 and 3.7±1.5 in the cupping therapy and control groups, respectively 2 weeks after the intervention and the results of repeated measures ANOVA revealed a significant difference between the two groups (P=0.001). Considering the short-form McGill pain questionnaire, the mean intensity of low back pain was 7.5±6.6 and 21.7±6.2 in the intervention and the control group, respectively 24 hours after the intervention. Also, it was measured as 3.6±4.1 and 14.0±5.2 in the cupping therapy and the control group, respectively 2 weeks after the intervention and the results of repeated measures ANOVA showed the difference to be statistically significant (P=0.001) (tables 1 and 2).

**Table 1:** Demographic characteristics and baseline values in the cupping therapy and control groups before the intervention

**Characteristics**	**Intervention group (n=50)**	**Control group (n=50)**	**P value**
Maternal age	25.0±4.2	27.0±3.8	0.064
Baseline values			
VAS	7.8±2.7	7.6±2.7	0.1
SMPQ	31.8±10.8	31.8±9.8	0.1
Sensory SMPQ	22.8±7.9	23.2±8.3	0.1
SMPQ emotional	9.0±3.4	9.2±2.9	0.1

*Values are expressed as mean±standard deviation. SMPQ: Short-form McGill Pain Questionnaire. VAS: Visual Analogue Scale

**Table 2:** Demographic characteristics and baseline values in the cupping therapy and control groups Immediately, 24 hrs, and 2 weeks post-intervention

**Measure**	**Phase**	**Intervention group**	**Control group**	**P value**
VAS	Baseline	7.8±2.7	7.6±2.7	0.1
	Immediately post-intervention	3.7±1.8	6.4±2.3	0.001
	24 hrs. post-intervention	2.5±1.7	5.0±2.0	0.001
	2 weeks post-intervention	1.4±1.4	3.7±1.5	0.001
SMPQ	Baseline	31.8±10.8	31.8±9.8	0.1
	Immediately post-intervention	9.0±6.7	29.2±8.0	0.001
	24 hrs. post-intervention	7.5±6.6	21.7±6.2	0.001
	2 weeks post-intervention	4.1±3.6	14.0±5.2	0.001
SMPQ sensory	Baseline	7.8±9.22	3.2±8.23	0.1
	Immediately post-intervention	5.0±5.7	9.8±5.21	0.001
	24 hrs. post-intervention	4.8±5.5	6.4±3.16	0.001
	2 weeks post-intervention	7.2±2.3	0.4±4.10	0.001
SMPQ emotional	Baseline	4.0±3.9	9.2±2.9	0.1
	Immediately post-intervention	1.1±2.2	8.5±2.7	0.001
	24 hrs. post-intervention	5.7±1.1	4.6±2.5	0.001
	2 weeks post-intervention	2.8±1.1	8.6±1.3	0.001

Note: SMPQ (Short-form McGill Pain Questionnaire) divided in two parts: sensory and emotional.

## Discussion

The present study was the first clinical trial on cupping therapy in gynecological diseases in Iran and aimed to investigate the effect of cupping therapy on the intensity of low back pain. Based on VAS, the mean intensity of low back pain reached from 7.8±2.7 before the intervention to 3.7±1.8, 2.5±1.7, and 1.4±1.4 immediately, 24 hours, and 2 weeks after the intervention, respectively and the results of repeated measures ANOVA showed a significant difference among different stages of follow-up (P=0.01). In addition, the corresponding measures in the short-form McGill pain questionnaire were 31.8±10.8, 9.0±6.7, 7.5±6.6, and 4.1±3.6, respectively (P=0.01). Overall, various theories have supported the advantages of cupping therapy. For instance, cupping therapy is believed to enhance the blood flow around the cup and help release the toxins trapped in the body tissues. Besides, it has been stated that this method moves discomfort from one place to another and, consequently, eliminates the pain.^26^ One study investigated the effectiveness of medical cupping therapy in 30 fibromyalgia patients in China. The researchers of that study used bamboo cups which were boiled in herbal plants for cupping. The results revealed a decrease in the score of pain compared to the beginning of the study (from 2.63±0.73 to 0.77±2.22 in 5 days and 1.78±0.75 in 10 days). Overall, cupping therapy led to reduction of pain and sensitivity in the patients suffering from fibromyalgia.^27^

Cupping therapy reduces local congestion through relative suction applied in the cups by heat or a sucker. This method has been employed for more than a thousand years. Although it is believed that cupping therapy originates from traditional Chinese medicine, this method is known as a beneficial treatment method all around the world.^28^

One study evaluated the effect of various branches of CAM, such as acupuncture, massage, and cupping therapy, in 68 patients with obstinate myofascitis and showed that a combination of these methods could lead to elimination of blood stagnation, improvement of blood circulation, energy channels enema, modification of metabolism, repair of tissues, and relaxation of the involved muscles.^29^ One year later (2008), the same researchers investigated the effect of cupping therapy at back-shu point on the chronic fatigue syndrome. In that study, 142 patients were recruited into the intervention group and underwent cupping therapy at both sides of the spinal cord. Then, the treatment effects were assessed using Fatigue Assessment Index (FAI). According to the results, the effect size was obtained as 97.9% in the intervention group and 79.6% in the control group (P<0.01). Moreover, a significant difference was found between the two groups’ FAI scores after the intervention (P<0.01).^30^

According to short-form McGill pain questionnaire in the present study, the mean intensity of the sensory dimension of low back pain in the intervention group reduced from 22.8±7.9 before the intervention to 7.0±5.5, 5.8±5.4, and 3.2±2.7 immediately, 24 hours, and 2 weeks after the intervention, respectively. Cupping therapy directs the blood flow towards the skin and muscles and stimulates the peripheral nervous system and, in this way, reduces the patients’ pain.^31^ On the other hand, the mean intensity of the emotional dimension of low back pain reached from 9.0±4.3 before the intervention to 2.1±2.1, 1.7±1.5, and 1.8±1.2 immediately, 24 hours, and 2 weeks after the intervention, respectively and the difference among the various stages of follow-up was statistically significant. Nevertheless, this measure increased 2 weeks after the intervention compared to 24 hours after that. This might be due to the disruption of the mothers’ relationship with the researcher. In fact, the psychological effects of cupping therapy which result from the consecutive status of the treatment as well as the close relationship between the therapists and the patients play a critical role in the emotional dimension of pain. These findings were consistent with those of a clinical trial in which acupuncture together with cupping therapy was done for Ankylosing Spondylitis (AS) in Chinese patients. According to the results, the rate of pain had clinically reduced by 62.5% in the intervention group and by 33.3% in the control group (P<0.01). In addition, the effect size was higher in the intervention group compared to the control group (93.8% vs. 83.3%; P<0.01). Besides, the rate of recurrence after 1 year was 3.3% and 24% in the intervention and the control group, respectively (P<0.01). Thus, the researchers concluded that the combination of acupuncture and cupping therapy was more effective in treatment of the disorder.^32^ In Chinese medicine, dry cupping therapy is performed after acupuncture at the same point; therefore, it is not surprising that the indications of cupping therapy are similar to those of acupuncture.^33^

In general, cupping therapy is performed at different body points in order to treat various disorders. The Korea Institute of Oriental Medicine (2012) for the first time assessed the effect of cupping therapy on neck pain in the video display terminals users. The results showed that cupping therapy was more effective compared to warm tissue (1.29 points reduction in the pain score through 3 weeks, P=0.025). Thus, it can be concluded that cupping therapy accompanied by exercising for 2 weeks was effective in reduction of neck pain and improvement of neck function in video display terminals users.^34^ In the same line, the findings of another study in Busan, South Korea indicated a significant difference between two groups of nurses regarding the frequency and intensity of shoulder pain and weakness after dry cupping therapy (P<0.05).^35^

In general, cupping therapy is more commonly used in Asian and East European countries and can eliminate pain or a series of symptoms, such as acute lumbar sprain. In Taiwanese traditional medicine, waist is considered as one of the most important parts of the body and in case it is damaged, various blood vessels and body meridians are blocked leading to acute pain and disability in movement. In Wang’s study, first the needles were applied to Houxi (SI3) and Shuigou (GV26) points. Afterwards, the needles were pulled out and cupping therapy was performed for 7 times each lasting for 3-5 minutes. The results revealed a significant difference between the two groups and the effect size was 100% and 80.6% in the intervention and the control group, respectively (P<0.05).^36^

Cupping therapy can be used either alone or in combination with other treatment methods. For instance, a study investigated the effect of laser acupuncture together with cupping therapy on low back pain in Taiwan. In that study, acupuncture was performed at BL40 point (Weizhong) using a laser with the wavelength of 808nm and frequency of 20Hz. Cupping therapy was also performed for 10 minutes. The data were gathered using VAS before and 5 consecutive days after the intervention. The VAS score reduced in both groups 5 days after the intervention, but no significant difference was found between the two groups during this period. On the second day for example, the intensity of pain was 2.11±4.60 and 2.12±5.09 in the intervention and the control group, respectively (P=0.182). Nonetheless, a significant difference was observed in the study groups concerning the pain intensity on the fifth day compared to the first day (P<0.01).^37^ Thus, these researchers managed to combine traditional and new medicine and treat one of the most prevalent pains. This emphasizes the comprehensiveness and richness of the traditional medicine, particularly acupuncture.

Overall, no severe complications have been mentioned for cupping therapy to affect its efficiency and safety. Furthermore, since dry cupping was utilized in the current study, the potential risk of blood-borne diseases by contaminated instruments was eliminated. 

One of the limitations of the present study was that the patients were aware of the mechanism and reason of the interventions and, consequently, the research could not be blinded. Nonetheless, due to the transparent nature of the study, this was out of the researcher’s control. 

## Conclusion

The study findings showed that dry cupping therapy at accupoint BL23 had a desirable effect on reduction of pain in the patients. It should be noted that the results of VAS in this study were quite in agreement with those obtained from short-form McGill questionnaire, while these two instruments measure different criteria of pain. The first one deals with the tangible intensity of pain, while the second one consists of the items which evaluate various sensory and emotional dimensions. Future studies are recommended to use herbal medicine as an analgesic combined with cupping therapy for relief of pain in obstetrics and gynecology domain. 
